# Economic Evaluation of PCSK9 Inhibitors in Reducing Cardiovascular Risk from Health System and Private Payer Perspectives

**DOI:** 10.1371/journal.pone.0169761

**Published:** 2017-01-12

**Authors:** Alejandro Arrieta, Timothy F. Page, Emir Veledar, Khurram Nasir

**Affiliations:** 1 Department of Health Policy and Management, Robert Stempel College of Public Health and Social Work, Florida International University, Miami, Florida, United States of America; 2 Center for Healthcare Advancement & Outcomes, Baptist Health South Florida, Miami, Florida, United States of America; 3 Miami Cardiac & Vascular Institute (MCVI), Baptist Health South Florida, Miami, Florida, United States of America; 4 Department of Medicine Herbert Wertheim College of Medicine & Department of Epidemiology, Robert Stempel College of Public Health and Social Work, Florida International University, Miami, Florida, United States of America; 5 The Johns Hopkins Ciccarone Center for the Prevention of Heart Disease, Baltimore, Maryland, United States of America; Royal College of Surgeons in Ireland, IRELAND

## Abstract

The introduction of *Proprotein covertase subtilisin/kexin type 9* (PCSK9) inhibitors has been heralded as a major advancement in reducing low-density lipoprotein cholesterol levels by nearly 50%. However, concerns have been raised on the added value to the health care system in terms of their costs and benefits. We assess the cost-effectiveness of PCSK9 inhibitors based on a decision-analytic model with existing clinical evidence. The model compares a lipid-lowering therapy based on statin plus PCSK9 inhibitor treatment with statin treatment only (standard therapy). From health system perspective, incremental cost per quality adjusted life years (QALYs) gained are presented. From a private payer perspective, return-on-investment and net present values over patient lifespan are presented. At the current annual cost of $14,000 to $15,000, PCSK9 inhibitors are not cost-effective at an incremental cost of about $350,000 per QALY. Moreover, for every dollar invested in PCSK9 inhibitors, the private payer loses $1.98. Our study suggests that the annual treatment price should be set at $4,250 at a societal willingness-to-pay of $100,000 per QALY. However, we estimate the breakeven price for private payer is only $600 per annual treatment. At current prices, our study suggests that PCSK9 inhibitors do not add value to the U.S. health system and their provision is not profitable for private payers. To be the breakthrough drug in the fight against cardiovascular disease, the current price of PCSK9 inhibitors must be reduced by more than 70%.

## Introduction

The introduction of *Proprotein covertase subtilisin/kexin type 9 (PCSK9)* inhibitors to the market has been heralded a major advancement. PCSK9 inhibitors significantly reduce low-density lipoprotein (LDL) cholesterol levels by about 47.5 percent [1,2] with no significant serious adverse events. Based on their efficacy and safety, the U.S. Food and Drug Administration (FDA) recently approved evolocumab [3] and alirocumab [4], two PCSK9 inhibitor drugs, for use in select individuals at high risk for cardiovascular disease (CVD). Although there is strong evidence supporting the efficacy of PCSK9 inhibitors in reducing LDL cholesterol, increasing high density lipoprotein (HDL) cholesterol, and decreasing total cholesterol [1,5]; their efficacy in reducing cardiovascular mortality and cardiovascular events is mixed and still inconclusive in the long-run [6]. In two open-label, randomized trials, Sabatine et al. [5] found a nearly 56 percent relative risk reduction in cardiovascular events after 1 year of therapy with evolocumab PCSK9 inhibitor plus standard therapy (statin with or without ezetimibe) in high-risk patients [5].

Despite enthusiasm regarding potential improvements in cardiovascular risk, genuine concerns have been raised on the added value to the health care system in terms of their cost and benefits [6,7]. The prices of the first two PCSK9 inhibitor drugs ranged between $14,100 and $14,600 per year [8], which raised concerns about the cost and benefits of PCSK9 inhibitors from the perspective of the U.S. healthcare system. In a recent economic evaluation, the Institute for Clinical and Economic Review simulated the use of the new drug among patients with heterozygous familial hypercholesterolemia (defined as patients with very high LDL cholesterol) and patients with atherosclerotic cardiovascular disease [9–11]. In both cases, the authors found that PCSK9 inhibitors were not cost-effective from a health system perspective. Contrarily, in a study by Amgen, producer of the PCSK9 inhibitor Repatha (evolocumab), the authors found the new drug was cost-effective when it was used among patients with heterozygous familial hypercholesterolemia, but not among patients with atherosclerotic cardiovascular disease [12]. In this study we use a different modelling approach to perform the cost-effectiveness analysis (CEA) of PCSK9 inhibitors from a health system perspective. Our results compare and shed light on the discrepancies found in the current CEA of PCSK9 literature.

However, the main contribution of our study is the additional business case analysis from the perspective of a private insurance payer. The payer perspective is relevant for the U.S. private, multipayer, insurance market, where return-on-investment (ROI) is an important reimbursement decision driver [13,14]. In contrast with national health systems where a societal perspective would be more relevant, in the U.S. insurance market not all benefits of PCSK9 inhibitors can be accrued by individual payers. Insurance companies fail to enjoy the long-term benefits of their investments in their beneficiaries’ health when members move to other insurance plans [15], and limit their benefits to avoided direct medical costs and to fixed premiums. Some studies have attempted to capture these characteristics by neglecting long-term benefits. For example, budget impact analysis only captures short-term benefits to reflect payer’s decision making [9–11], and observational cost analysis of preventing major adverse CVDs only focuses on short-term available data [16]. While there is consensus that health insurance payers put less weight to long-term health benefits, it is unrealistic to assume that such weights are zero.

Our study addresses three questions: 1) Are PCSK9 inhibitors cost-effective from a health system perspective? 2) Do PCKS9 inhibitors generate a positive ROI for private insurance payers? 3) At what price would PCSK9 inhibitors add a positive net benefit to the health system and private payers? Although many therapies covered by insurance are not cost-saving, this analysis will determine the price at which PCSK9 inhibitors would produce a positive ROI. At or below this price, PCSK9 inhibitors would likely be integrated into clinical practice guidelines and become the standard prescribed treatment. Prices above this threshold would require insurance companies to increase premiums to their members in order to recover financial losses.

## Methods

### Study Population

The model considers a hypothetical cohort of patients beginning at age 58-year followed until death or age 100. Their baseline characteristics resemble those of the evolocumab PCSK9 inhibitor trial population [5], where about 70% were under statin treatment. In our hypothetical cohort, 52% of participants were male, 88% were currently on medication to treat high blood pressure, 16% were smokers and 13% had diabetes. Average systolic blood pressure was 128 mm/Hg for those under any antihypertensive medication, and 133 mm/Hg for those who were not under treatment. At baseline, the average HDL cholesterol was 51 mg/dL, LDL cholesterol was 120 mg/dL and total cholesterol was 202 mg/dL. All of these parameters were obtained from the evolocumab trial [5].

### Decision-Analytic Model

**Fig 1:**
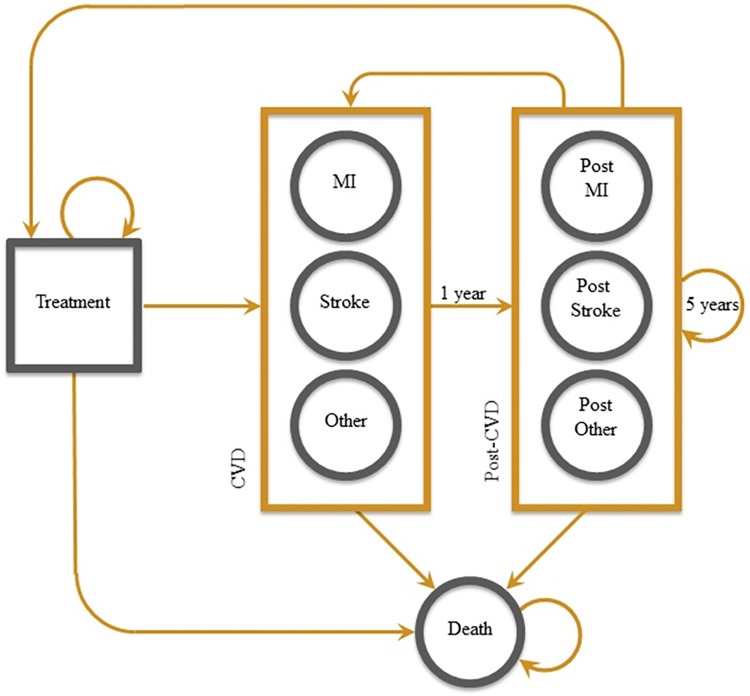
Decision-analytic model describing treatment and CVD events. Treatment could be PCSK9 inhibitor or standard-therapy. MI: Myocardial Infarction. Other: Other CVD event including unstable angina, transient ischemic attack, congestive heart failure, etc.

We developed a Markov decision-analysis model to compare a lipid-lowering therapy based on: A) statin plus PCSK9 inhibitor treatment, with B) statin treatment only (standard therapy). In the model (Fig 1) patients may live a normal life under lipid-lowering treatment or may develop a myocardial infarction, stroke, or other CVD event (unstable angina, transient ischemic attack, congestive heart failure, etc.). After the CVD event, patients move to a post-CVD stage that characterizes the slow transition back to normal life in terms of quality of life and costs. Patients may remain in the post-CVD stage for up to five years. They may have a subsequent CVD event. They may have a CVD-related death. Patients transition among stages on a 1-year cycle, and may die for any non-CVD-related cause at any stage of the Markov model. All simulations were performed in Microsoft Excel.

### Data Sources and Parameters

The parameters used in the model for the probability of a CVD event, mortality, treatment cost, and health utility states are presented in Table 1. Further details about the model parameters and assumptions are provided in S1 Appendix.

**Table 1:** Model Parameters.

Input Variable	Parameter	(Range or S.E.)	Source
Rates and Probabilities			
1-year probability of CVD event			
From Framingham study			
MI	0.14%-1.16%	(age dependent)	D’Agostino et al.
Stroke	0.08%-0.63%	(age dependent)	D’Agostino et al.
Other CVD	0.23%-1.65%	(age dependent)	D’Agostino et al.
From Sabatine et al. study			
MI	0.30%-1.68%	(age dependent)	Sabatine et al.
Stroke	0.05%-0.28%	(age dependent)	Sabatine et al.
Other CVD	0.25%-1.40%	(age dependent)	Sabatine et al.
1-year probability of sub-sequent CVD event			
MI	0.48%-1.86%	(age dependent)	D’Agostino et al.
Stroke	0.32%-1.02%	(age dependent)	D’Agostino et al.
Other CVD	0.93%-2.83%	(age dependent)	D’Agostino et al.
Mortality rates			
After MI	8.2%-98.5%	(age dependent)	CDC
After Stroke	5.9%-93.4%	(age dependent)	CDC
After other CVD	7.0%-96.0%	(age dependent)	CDC
After post-MI	3.6%-67.3%	(age dependent)	Bronnun et al.
After post-Stroke	3.3%-60.9%	(age dependent)	Bronnun et al.
After post-other CVD	3.4%-64.1%	(age dependent)	Bronnun et al.
Non CVD related	0.5%-18.3%	(age dependent)	CDC
Costs			
Statin treatment	$48	($36–$60)	Erickson et al.
PCSK9 treatment	$12,048	($9,036–$15,060)	
Baseline treatment	$215	($161–$269)	Davis et al.
MI	$11,071	($8,303–$13,839)	Erickson et al.
Post-MI	$7,747	($5,810–$9,684)	Erickson et al.
Stroke	$18,516	($13,887–$23,145)	Erickson et al.
Post-Stroke	$7,770	($5,828–$9,713)	Erickson et al.
Other CVD	$14,794	($11,095–$18,492)	Erickson et al.
Post–other CVD	$7,759	($5,819–$9,698)	Erickson et al.
Utility			
Statin/PCSK9 treatment	0.79		Sullivan at al.
MI (range over 5 years)	0.58–0.79	(0.54, 0.91)	Sullivan at al.
Stroke (range over 5 years)	0.46–0.79	(0.52, 0.87)	Sullivan at al.
Other CVD (range over 5 years)	0.63–0.79	(0.57, 0.96)	Sullivan at al.
Dead	0		
Health insurance parameters			
Premium			
Age			
58–64	$223–$520	(age dependent)	eHealth 2014
65+	$835	($751.6, $1,043.9)	CMS 2014
Deductible			
Age			
58–64	$3,882–$3,731	(age dependent)	eHealth 2014
65+	$2,158	($1,942.2, $2,697.5)	CMS 2014
Tier 1			
Age			
58–64	11	(9.9, 13.75)	eHealth 2014
65+	5	(4.5, 6.25)	CMS 2014
Tier 2			
Age			
58–64	31	(27.9, 38.75)	eHealth 2014
65+	11	(9.9, 13.75)	CMS 2014
Turnover rate	12.20%	(0.71%)	Cutler et al.

A key parameter in the model is the annual probability of CVD events over the patient lifespan. For our baseline scenario we projected annual probabilities at age 58 and beyond using the one year relative risk reduction from the evolocumab study [5] and the baseline survival function from the 10-year Framingham study [17], under the assumption that the Framingham survival function is proportional to the unobserved evolocumab survival function. The one year relative risk reduction of CVD events, excluding non-CVD deaths and coronary revascularization, was estimated at -49.2%.

### Health System Perspective

A Cost-Effectiveness Analysis (CEA) was performed from a health system perspective. We considered the cost of the lipid-lowering treatment under therapies A and B, and the avoided healthcare costs (savings) associated with reduced future CVD events that result from lowering LDL cholesterol levels. The quality adjusted life years (QALYs) and costs of CVD events are presented in Table 1. QALYs were obtained from the EQ-5D index score for chronic conditions in the U.S. [18] QALYs at treatment were estimated at 0.79, which corresponds to the relatively symptomatic cohort of 58-year-old hypothetical patients whose baseline characteristics resemble those of the evolocumab PCSK9 inhibitor trial population (hypertension, diabetes, and lipoid metabolism). For the 5 years in the post-CVD event, we assume a non-linear progression from the low 25% EQ-5D QALY score to the QALY at treatment. All QALYs were age-dependent. Costs are discussed below.

The incremental cost-effectiveness ratio (ICER) was defined as the difference between therapies A and B in treatment costs less any avoided healthcare costs from CVD events divided by the difference in QALYs between therapies A and B. This yielded the net cost per QALY improvement for PSCK9 inhibitors compared to standard therapy. Both, costs and QALYs were discounted at a 3% rate.

### Payer Perspective

A ROI analysis was also performed from a private insurance payer perspective. Three elements characterize this perspective. First, individual payers do not accrue all the long-term benefits of their investment because a fraction of members switch plans every year. A turnover rate of 12% was assumed for insurance membership i.e. 12% of individuals move to other insurance plans each year [19].

Second, the main source of benefit is the premium income stream, which is independent of the health status of the beneficiary (except in the case of death status). We considered average insurance premiums from private individual health insurance plans when members were 57–64 years old. When they turned 65, they were assumed to be in a Medicare Advantage plan. We assumed a monthly premium of $520 for age 57–64 years old [20], and $835 for 65 or older [21].

Third, private payer plans have a cost-sharing component. It was assumed the insurance payer pays for therapy A or B after copayments and deductibles are paid by the patient. For statins, payment coverage as a tier 1 drug and for PCSK9 inhibitors as a tier 2 drug was assumed. No copayments/coinsurance for visits to providers were assumed. We considered average insurance drug copayment and deductible from private individual health insurance plans when individuals were 57–64 years. Drug monthly copayments were assumed at $11 for statins and $31 for PCSK9, and an annual deductible of $3,731 was assumed for this age group [22]. For individuals 65 years old or older, we considered average Medicare Advantage plan cost-sharing. We assumed a drug monthly copayment of $5 for statins and $11 for PCSK9, and an annual deductible of $2,158 for this age group [21].

The net present value (NPV) to the private payer was the avoided costs plus the insurance premium less the cost of treatment, all at present values using a discount rate of 3%. Therefore, the ROI is the NPV divided by the treatment cost. An ROI of less than zero would indicate a net loss to the payer, while a ROI of greater than zero would indicates a net monetary gain to the payer.

### Costs

The cost of statin treatment (standard therapy) was assumed at $48 per year based on a low-cost generic therapy [23,24]. The price of PCSK9 inhibitors was assumed at $14,000 per year based on recent market reports [8]. Annual baseline treatment cost assumed one regular office-based physician visit per year at $215 [25].

Costs of myocardial infarction and stroke are based on Medicare claims and clinical trial studies, and they were obtained from Erikson and coauthors [23] as described in their technical appendix. The cost of myocardial infarction was assumed at $11,071 and at $7,647 the next year after the event. Cost of stroke was assumed at $18,516 and at $7,770 the next year after the event [23]. For years 2 to 5 after the CVD event, we assume a reduction of 25% in the cost from previous year. For other CVDs, we took the average cost of myocardial infarction and stroke.

### Uncertainty and Price Sensitivity Analysis

To account for the uncertainty in model parameters, an assumption was made about the probability distribution of costs, QALYs, insurance deductibles, copayments and premiums, and transition probabilities of the Markov model. First, our baseline scenario for annual probabilities of CVD events was changed. This alternative scenario was based on the 10-year Framingham risk equation [17] evaluated for the average patient from the evolocumab study. In this scenario, annual probabilities of CVD events were obtained indirectly by imputing the changes in LDL cholesterol reported by the evolocumab study (61% reduction in LDL level) into the Framingham equation.

Second, a sensitivity analysis to price variability was proposed to PCSK9 inhibitors. Different scenarios of PCSK9 inhibitor prices ranging from $500 to $15,000 a year were considered. For each PCSK9 inhibitor price scenario, 1,000 independent Monte Carlo simulations of costs and QALYs based on the probability distribution assumptions were drawn. Costs and QALYs were drawn from a beta distribution, insurance turnover rate was draw from a normal distribution. Probabilities of CVD and subsequent CVD events were drawn for each coefficient of the corresponding risk equation, assuming a normal distribution. An ICER was obtained for each simulation, and all ICERs were compared to thresholds to produce acceptability curves [26] for each price scenario. For the health system perspective, each ICER was compared to willingness-to-pay thresholds of $20,000, $50,000 and $100,000 for each additional QALY gained.

In further analyses, the acceptability of different price scenarios from the health system and the private insurance payer perspectives was obtained. From a payer perspective, the acceptability is given by the probability of a positive ROI at each price level. All results are presented in the form of acceptability curves.

## Results

### CEA and ROI Analysis

Table 2 presents the overall cost increase, compared to standard therapy, of PCSK9 inhibitors at $14,000 and $15,000 annual price. It also shows the avoided costs or savings due to reduced CVD events. Treatment and avoided costs are from the health system’s and payer’s perspective. Payer’s premium revenues are also presented as well as overall changes in QALYs, life-years, and CVD events.

**Table 2:** Incremental Costs, Revenues and Outcomes with PCSK9 (Per Patient. Health System’s and Payer’s Perspective) ^1/^.

	Perspective	
	Health System	Payer ^2/^
Treatment cost (If price = $14,000)	$237,718	$73,137
Treatment cost (if price = $15,000)	$254,695	$78,463
Avoided cost (savings)	-$5,800	-$1,095
Premium revenue		$644
QALY	0.66	
Life years	0.88	
CVD events	-0.41	

^1/^ Effect of PCSK9 on annual probability of CVD is obtained directly from the 1 year effect study of evolocumab.

^2/^ Private insurance perspective assuming national average premiums, medication copayments and deductibles. It also includes a health insurance turnover rate of 12%.

All costs, revenues and outcomes are per patient and discounted at 3% discount rate.

Table 3 summarizes the ICER and ROI results across different price scenarios. The current $14,000 price point yields an ICER of $348,807 per QALY. Considering the highest willingness-to-pay threshold of $100,000 per QALY, then PCSK9 is cost-effective from a health system perspective only if the price is less than or equal to $4,250. However, at the cost-effective price of around $4,250, PCSK9 is still not financially viable for private insurers, since they face losses of $19,506 per treated patient, equivalent to a -91.8% ROI. PCSK9 is only financially viable for payers (and health system) at a price of around $600 per year.

**Table 3:** CEA and ROI analysis at Different Prices of PCSK9 (Health System’s and Payer’s Perspective) ^1/^.

PCSK9 inhibitor price	Perspective		
	Health System	Payer ^2/^	
	ICER	ROI	NPV
$500	$4,103	39.8%	$495
$1,000	$16,870	-55.5%	-$2,167
$2,500	$55,170	-85.4%	-$10,156
$5,000	$119,004	-93.1%	-$23,469
$7,500	$182,838	-95.5%	-$36,783
$10,000	$246,673	-96.6%	-$50,096
$12,500	$310,507	-97.3%	-$63,410
$14,000	$348,807	-97.6%	-$71,398
$15,000	$374,341	-97.8%	-$76,723
Breakeven prices			
$593	$6,478	0.0%	$0
$4,256	$100,000	-91.8%	-$19,506

^1/^ Effect of PCSK9 inhibitors on annual probability of CVD is obtained directly from the 1 year effect study of evolocumab.

^2/^ Private insurance perspective assuming national average premiums, medication copayments and deductibles. It also includes a health insurance turnover rate of 12%.

ICER = Incremental cost-effectiveness ratio. ROI = Return on investment. NPV = Net present value.

### Uncertainty and Price Sensitivity Analysis

Changing our scenario of the effect of PCSK9 inhibitors on the one year relative risk reduction in CVD events affects the cost-effectiveness and ROI results. When the reduction in CVD events are obtained through the 10-year Framingham risk equation, PCSK9 inhibitors become less cost-effective and less profitable to private insurers. Under this alternative scenario, the $14,000 price point yields an ICER of $617,542 per QALY, and losses to private insurers of $72,504 per patient (see S1 Appendix).

**Fig 2:**
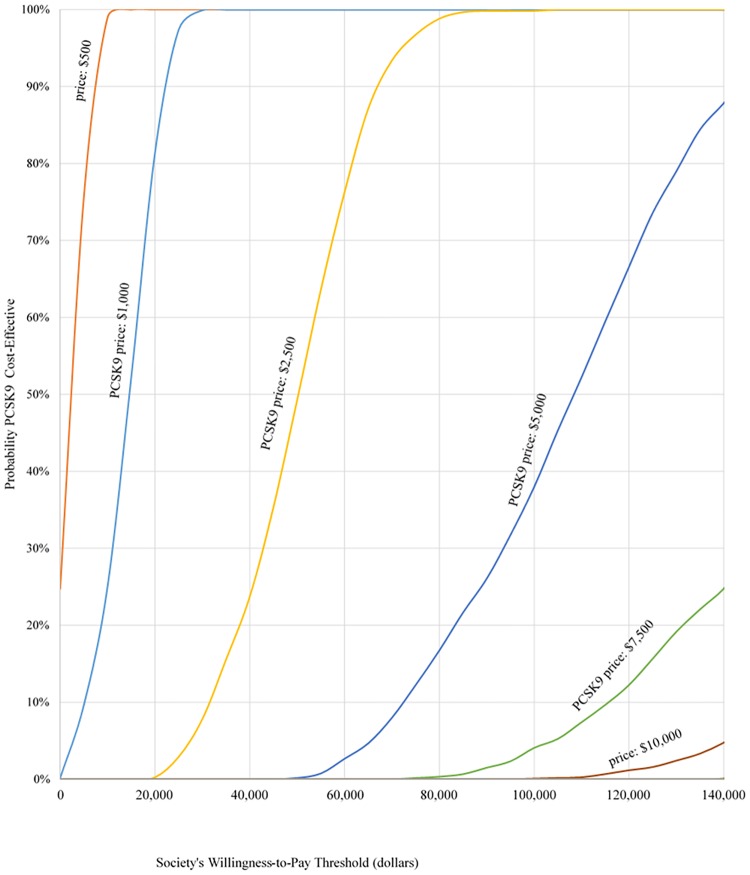
Acceptability curve from health system perspective.

Acceptability curves under baseline scat different price scenarios are shown in Fig 2. For a willingness-to-pay threshold of $100,000 per QALY, a PCSK9 inhibitor therapy priced at $2,500 per year will be cost-effective about 99.8% of the time. A price of $5,000 will be cost-effective 38% of the time, and a price of $7,500 will be cost-effective 4% of the time. At the current market price of $14,000, there is a zero probability of PCSK9 inhibitors being cost-effective.

**Fig 3:**
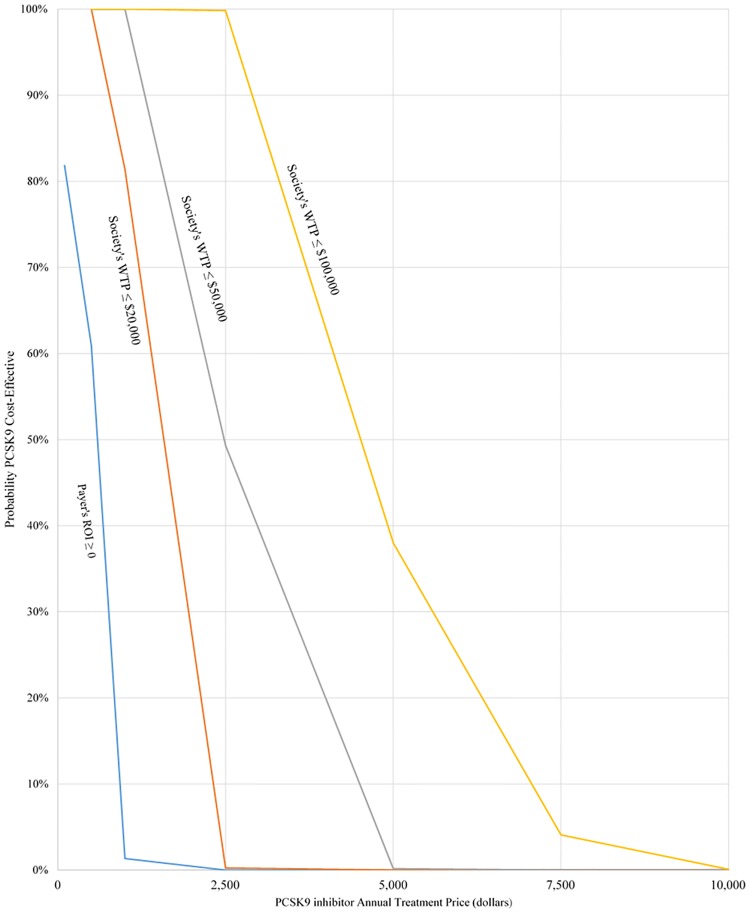
Acceptability from private and health system perspective.

Acceptability of PCSK9 inhibitors from private and health system perspective at different PCSK9 inhibitors price scenarios is presented in Fig 3. The figure represents the probability that PCSK9 inhibitor treatment adds value to the health system and an average private payer, at different PCSK9 prices. At a price of $2,500 a year, a PCSK9 inhibitor has a high probability of being cost-effective from a health system perspective (99.8% at a willingness-to-pay threshold of $100,000 per QALY and 49.3% at $50,000 per QALY). However, at that same price of $2,500, the probability that an average private payer obtains a positive ROI is nearly zero. Only at a price around $600 or less the average private payer would break even.

## Discussion

Our study results suggest that from a health system perspective, PCSK9 inhibitors are not cost-effective at current prices. At the current $14,000 to $15,000 price, the incremental cost per additional QALY produced is nearly three and a half times the usual societal acceptable $100,000 threshold. Moreover, our study finds that PCSK9 inhibitors are not financially viable for an average private insurance payer. The ROI analysis, relevant for the U.S. multipayer insurance market, suggests that the price of PCSK9 inhibitors would have to receive large discounts to be a financially sound investment for insurers. Without price reductions, insurers would have to recover the losses of covering these drugs by charging higher premiums. Because insurers can no longer charge higher premiums to individuals with chronic health conditions under the terms of the Affordable Care Act, this burden would be spread over all insured patients. However, it is unlikely that this cost would be fully transferred to premiums given the rate-review provisions in the Affordable Care Act, which may result in increased barriers to accessing these new drugs.

While robust LDL reduction has been established with the novel PCSK9 inhibitors, outcome benefits of PCSK9 inhibitors have not yet been documented [1,5,27]. Under the assumption of maximal improvement in CVD of 49.2% relative risk reduction based on the open label studies, our results reveal that at current prices, PCSK9 inhibitors are unlikely to be cost-effective from a health system perspective nor profitable for insurers. At the current price of $14,000 to $15,000, for every dollar invested, private insurers lose $1.98. For insurers to break-even, the price would have to fall to $600. To be cost-effective from a health system perspective, the price would have to be reduced to $4,250.

Our results from a health system perspective compare to two recent CEA of PCSK9 inhibitors. The Institute for Clinical and Economic Review estimated an ICER of $503,000 per QALY [9–11], which is higher than our baseline estimate. Contrarily, researchers from Amgen, producer of the PCSK9 inhibitor Repatha (evolocumab), estimated an ICER of $75,800 per QALY [12], which is lower than our baseline estimate. The main reason explaining the large difference in ICER estimates is the assumption in the dose-response between LDL cholesterol and CVD events. In sensitivity analyses conducted to account for uncertainty in the study parameters, drug prices, and societal willingness-to-pay for QALY improvements, we observed cost-effectiveness and ROI to be highly sensitive to assumptions about the effect of PCSK9 inhibitors on CVD events. All savings associated to PCSK9 inhibitors are the result of avoided CVD events and mortality in the future. From a modelling point of view, reductions in LDL cholesterol add value only if they are able to reduce CVD events. Since evidence about the efficacy in reducing CVD events is mixed and still inconclusive in the long-run [6], this assumption represents the major limitation of economic evaluation studies of PCSK9 inhibitors. To incorporate the uncertainty of this parameter, we not only performed a sensitivity analysis, but also considered an alternative scenario for the dose-response assumption between LDL cholesterol and CVD events. Our baseline scenario combined the evolocumab study results with the 10-year Framingham risk equation to estimate the long-run effect on CVD events, which allows for a more realistic age-dependent dose-response between cholesterol levels and CVD events. Our alternative scenario related LDL cholesterol levels with CVD events through the Framingham risk equation, producing an ICER of $617,542 per QALY. Overall, our results from a health system perspective coincide with the Institute for Clinical and Economic Review’s conclusion that PCSK9 inhibitors are not cost-effective at current prices.

Our main limitation is that the effects of PCSK9 inhibitors on CVD outcomes are not yet know. Studies are still limited to one-year randomized trials, and they are silent to long-run effects. Although we reduced this limitation by considering two scenarios for annual probabilities of CVD events in the long-run, our results are still limited to the lack of actual data on effectiveness. As with any other decision analytic model, our methodology is also limited to the simplifying assumptions about the underlying cohort characteristics used in analysis, which do not represent the U.S. population. This study applies to a hypothetical population that resembles the participants in the evolocumab PCSK9 study. What we lose in terms of representing a larger population, we gain in terms of more accurate assumptions for the specific cohort. We share the limitations of CEA, an incomplete tool that does not incorporates all of a drug's salient characteristics [28].

In conclusion, our study suggests that at current prices, PCSK9 inhibitors are unlikely to add value to either the health system or private insurance payers. To be the breakthrough drug in the fight against cardiovascular disease, the current price must be reduced to make PCSK9 inhibitors cost-effective to the health system and financially viable to payers. Our study also finds an important gap between the price at which PCSK9 inhibitors become cost-effective and the break-even price for private payers. This gap reflects the complexity of implementing a value-based payment system in the U.S. healthcare market, and the challenges of aligning right financial incentives for private payers to cover for cost-effective drugs. Because insurance companies face all the investment cost but only a fraction of the long-term benefits, the payer’s private value is generally lower than health system’s value. While payers can increase private value by transferring the investment cost through higher cost-sharing to patients (for example, listing PCSK9 inhibitors as tier 3 or 4 drugs), or higher premiums to all members, the current high price of PCSK9 inhibitors and the provisions of the Affordable Care Act may limit these price transfer mechanisms. A major concern is that with limited price reduction or transferred options, payers limit the access to the new drug. Given that the efficacy of PCSK9 inhibitors on reducing CVD events is still inconclusive, and that ICER estimates are highly sensitive to this assumption, limiting utilization of the drug may penalize a potential high-value health technology [15]. A promising reimbursement strategy that is gaining attraction in the U.S. are outcome-based contracts that tie price to patient outcomes. A recent survey from Avalere [29] reports that a few insurance companies have entered in outcome-based contracts with producers of PCSK9 inhibitors in which they receive large discounts if LDL cholesterol reductions among patients are lower than those observed in clinical trials. This type of outcome-based reimbursement transfers some financial risk from payers to pharmaceutical companies, which may help high-value drugs and benefit patients.

## Supporting Information

S1 Appendix Data Sources, Parameters and Additional Results. (DOCX)
