# Supplementary material for: Economic Evaluation of PCSK9 Inhibitors in Reducing Cardiovascular Risk from Health System and Private Payer Perspectives
Source: PLoS One. 2017 Jan 12;12(1):e0169761. doi: 10.1371/journal.pone.0169761 (PMC5232345; doi:10.1371/journal.pone.0169761)
Supplement: S1 Appendix — (DOCX) [file pone.0169761.s001.docx]

**Supporting Information**

# S1 Appendix. Data Sources, Parameters and Additional Results

1. The transition probabilities of our Markov model were age-dependent. The transition of a cohort under treatment A (statin plus PCSK9 inhibitor) was compared with an identical cohort under treatment B (standard therapy). Probabilities were obtained from the literature and adjusted to the model as follows (letters correspond to S1 Fig):

**S1 Fig. Annotated Decision-Analytic Model**


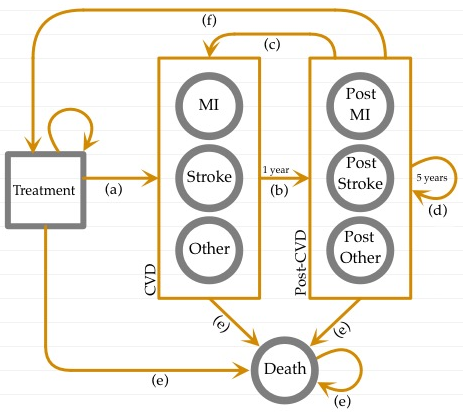


1. 1-year probability of CVD event at ages 58 to 100.
   - For our baseline scenario we used the one year relative risk reduction of CVD events reported in the evolocumab study[^1^](#_ENREF_1), excluding non-CVD deaths and coronary revascularization (obtained from Supplemental Table 4 in Sabatine et al.) To project annual probabilities of CVD events at age 59 to 100 we used the baseline survival function from the 10-year Framingham study,[^2^](#_ENREF_2) under the assumption that the Framingham survival function is proportional to the unobserved evolocumab survival function.
   - An alternative scenario was based on the 10-year Framingham risk equation[^2^](#_ENREF_2) evaluated for the average patient from the evolocumab study[^2^](#_ENREF_2). In this scenario, 1-year probabilities of CVD events were obtained indirectly by imputing the changes in LDL cholesterol reported by the evolocumab study in the Framingham equation.
   - In all scenarios, 1-year probabilities of CVD events were obtained separately for males and females for each CVD event (MI, stroke and other CVD) based on the 2008-2010 average of CVD distribution by age[^3^](#_ENREF_3). Probabilities were then combined based on female-male distribution.
   - 1-year probabilities of CVD events for ages 58 to 100, for our baseline and alternative scenarios are represented in S2 Fig.

**S2 Fig. One-year Probabilities of CVD Events**


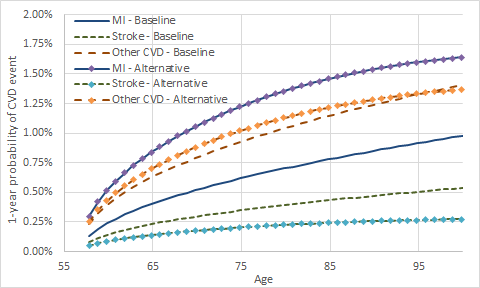


1. Transition to post-CVD event occur after one cycle (one year) with probability 1 (tunnel state).
2. 1-year probability of subsequent CVD events at ages 58 to 100. Probabilities were obtained from 4-year follow-up Framingham study on subsequent CVD events[^4^](#_ENREF_4). 1-year probabilities of CVD events were obtained separately for males and females for each CVD event (MI, stroke and other CVD) based on the 2008-2010 average of CVD distribution by age[^3^](#_ENREF_3). Probabilities were then combined based on female-male distribution. 1-year probabilities of CVD events were obtained separately for males and females for each subsequent CVD event (MI, stroke and other CVD) based on the 2008-2010 average of CVD distribution by age[^3^](#_ENREF_3). Probabilities were then combined based on female-male distribution. S3 Fig shows the 1-year probabilities of subsequent CVD events for ages 58 to 100.

**S3 Fig. One-year Probabilities of Subsequent CVD Events**


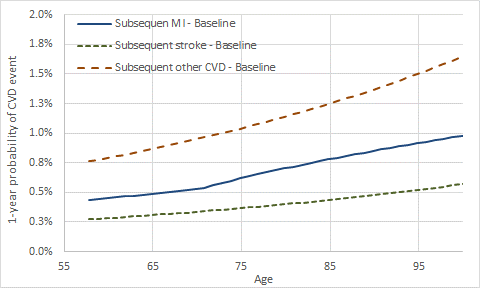


1. Progression within post-CVD event (tunnel state). Individuals are assumed to remain in the post-CVD event for 5 years, implying that it takes 5 years to recuperate from the CVD event. At any time during that 5-year period, they can transit to a subsequent CVD event (c) or they can die (e).
2. Transition to death (absorbing state).
   - Mortality probabilities after a MI and post-MI event and after a stroke and post-stroke event were obtained from Bronnum et al.[^5^](#_ENREF_5)^,^ [^6^](#_ENREF_6).
   - Non-CVD mortality was obtained from the 2010 U.S. life tables[^7^](#_ENREF_7). Overall CVD mortality was calibrated using clinical characteristics of the general population to match the life expectancy of the U.S. population.
3. Transition to treatment (absorbing state).
4. Additional results using one-year probability of CVD events obtained from Framingham study are presented in S1 Table.

**S1 Table. Cost Effectiveness at Different Prices of PCSK9 (Health System’s and Payer’s Perspective) ^1/^**

|  | Perspective | | |
| --- | --- | --- | --- |
| PCSK9 inhibitor price | Health System | Payer ^2/^ | |
|  | ICER | ROI | NPV |
| $500 | $14,523 | -30.46% | -$381 |
| $1,000 | $36,857 | -77.83% | -$3,052 |
| $2,500 | $103,859 | -92.72% | -$11,066 |
| $5,000 | $215,529 | -96.56% | -$24,422 |
| $7,500 | $327,199 | -97.75% | -$37,778 |
| $10,000 | $438,869 | -98.33% | -$51,134 |
| $12,500 | $550,540 | -98.67% | -$64,491 |
| $14,000 | $617,542 | -98.82% | -$72,504 |
| $15,000 | $662,210 | -98.90% | -$77,847 |

1/ Effect of PCSK9 on annual probability of CVD is obtained indirectly through the LDL effect using the Framingham risk equation.

2/ Private insurance perspective assuming national average premiums, medication copayments and deductibles. It also includes a health insurance turnover rate of 12%.

ICER=Incremental cost effectiveness ration. ROI=Return on investment. NPV=Net present value.

**References**

1. Sabatine MS, Giugliano RP, Wiviott SD, Raal FJ, Blom DJ, Robinson J, Ballantyne CM, Somaratne R, Legg J, Wasserman SM. Efficacy and safety of evolocumab in reducing lipids and cardiovascular events. *New England Journal of Medicine*. 2015;372:1500-1509

2. D’Agostino RB, Vasan RS, Pencina MJ, Wolf PA, Cobain M, Massaro JM, Kannel WB. General cardiovascular risk profile for use in primary care the Framingham Heart Study. *Circulation*. 2008;117:743-753

3. CDC. Health Data Interactive (2008-2010).

4. D'Agostino RB, Russell MW, Huse DM, Ellison RC, Silbershatz H, Wilson PW, Hartz SC. Primary and subsequent coronary risk appraisal: new results from the Framingham study. *American Heart Journal*. 2000;139:272-281

5. Brønnum-Hansen H, Davidsen M, Thorvaldsen P, Group ftDMS. Long-Term Survival and Causes of Death After Stroke. *Stroke*. 2001;32:2131-2136

6. Brønnum-Hansen H, Jorgensen T, Davidsen M, Madsen M, Osler M, Gerdes LU, Schroll M. Survival and cause of death after myocardial infarction: the Danish MONICA study. *Journal of Clinical Epidemiology*. 2001;54:1244-1250

7. CDC. CDC Health Data Interactive.
